# Crystal structure of bis­(propane-1,3-diaminium) hexa­fluorido­aluminate di­aqua­tetra­fluorido­aluminate tetra­hydrate

**DOI:** 10.1107/S1600536814024155

**Published:** 2014-11-08

**Authors:** Insaf Abdi, Khulood Al-Sadhan, Amor Ben Ali

**Affiliations:** a Université de Carthage, Faculté des Sciences de Bizerte, UR11ES30, 7021 Jarzouna, Tunisia; b Department of Chemistry, Girls College of Science, University of Dammam, PO Box 838, Dammam 31113, Saudi Arabia

**Keywords:** crystal structure, hybrid organic–inorganic fluoride compound, fluorido­aluminate, aluminium, hydro­thermal synthesis, hydrogen bonding

## Abstract

The title compound, (C_3_H_12_N_2_)_2_[AlF_6_][AlF_4_(H_2_O)_2_]·4H_2_O, was obtained by a solvothermal method in ethanol as solvent and with aluminium hydroxide, HF and 1,3-di­amino­propane as educts. The asymmetric unit contains a quarter each of two crystallographically independent propane-1,3-di­ammonium dicat­ions, [AlF_6_]^3−^ and [AlF_4_(H_2_O)_2_]^−^ anions and four water mol­ecules. The cations, anions and three of the independent water mol­ecules are situated on special positions *mm*, while the fourth water mol­ecule is disordered about a mirror plane. In the crystal, inter­molecular N—H⋯F and O—H⋯F hydrogen bonds link the cations and anions into a three-dimensional framework with the voids filled by water mol­ecules, which generate O—H⋯O hydrogen bonds and further consolidate the packing.

## Abstract 2

In the crystal structure of bis­(propane-1,3-di­ammonium) hexa­fluorido­aluminate di­aqua­tetra­fluorido­aluminate tetra­hydrate, two different environments of the Al^3+^ cations are observed, namely, AlF_6_ and AlF_4_(H_2_O)_2_.

## Chemical context   

Hybrid organic–inorganic fluoride compounds are composed of both organic and inorganic moieties. The search for new compounds in this class of materials is still intense due to their applications in many domains such as gas storage, catalysis, separation, ion-exchange and biomedicine (Horcajada *et al.*, 2012 ▶; Stock & Biswas, 2012 ▶). Various hybrid materials containing fluorine organic ligands have been described in the literature (Ben Ali *et al.*, 2007 ▶). The dimensionality of the metal fluoride entities are 0D (isolated polyanions) (Adil, Ben Ali *et al.*, 2006 ▶; Adil, Leblanc & Maisonneuve, 2006 ▶; Fourquet *et al.*, 1987 ▶) , 1D (chains) or 2D (layers) (Adil *et al.*, 2010 ▶). The structural architecture of hybrid materials mainly depends on the metal and an organic part. However, other physical and physicochemical factors affect the resulting products such as the synthesis method (temperature, concentration, time of heating *etc*.) (Su *et al.*, 2010 ▶). This work is a continuation of an exploration of chemical systems including metal fluoride and amine, and the study of their structures.
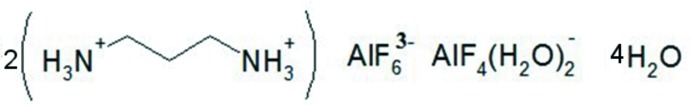


## Structural commentary   

**Figure 1:**
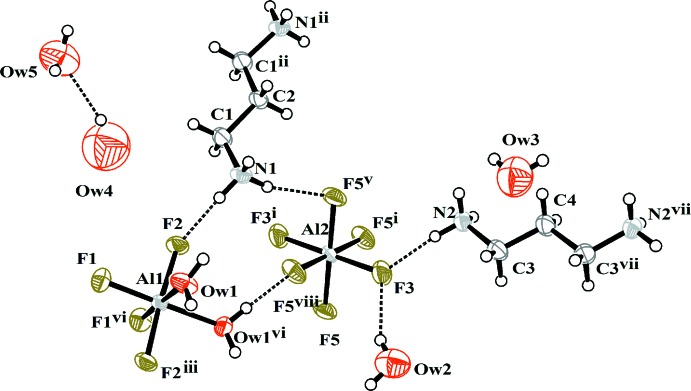
A portion of the crystal structure of the title compound showing the atom labelling and 50% probability displacement ellipsoids. Dashed lines denote hydrogen bonds. [Symmetry codes: (i) 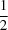 − *x*, 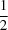 − *y*, −1 − *z*; (ii) 1 − *x*, *y*, *z*; (iii) *x*, *y*, −*z*; (v) 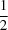 − *x*, 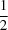 − *y*, 1 + *z*; (vi) −*x*, *y*, *z*; (vii) *x*, −*y*, −1 − *z*; (viii) *x*, *y*, 1 + *z*.]

The asymmetric unit of the title compound contains aluminum atoms located in two crystallographically independent sites with different environments, [Al2F_6_] and [Al1F_4_(H_2_O)_2_], and two independent 1,3-propane di­amine (dap) dications (Fig. 1 ▶). The Al—F distances in the two octa­hedra range from 1.768 (2) to 1.809 (3) Å while the Al1—O*W*1 distance is longer [1.944 (4) Å]. The [AlF_6_] octa­hedron is regular whereas [AlF_4_(H_2_O)_2_] exhibits a pronounced distortion due to the strong influence of the crystal field created by the heteroligands (F^−^/H_2_O). The value of the calculated valences (3.08 for Al1 and 3.01 for Al2) of the individual Al^3+^ cations (Brese & O’Keeffe, 1991 ▶) is in good agreement with the theoretical value, whereas those for the F^−^ anions are equal to 0.5. These anions complete their valence by establishing strong hydrogen bonds.

## Supra­molecular features   

**Figure 2:**
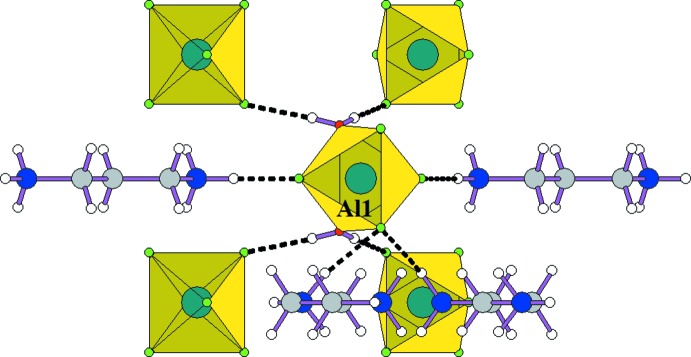
The environment of the AlF_4_(H_2_O)_2_ octa­hedron. Dashed lines denote hydrogen bonds.

**Figure 3:**
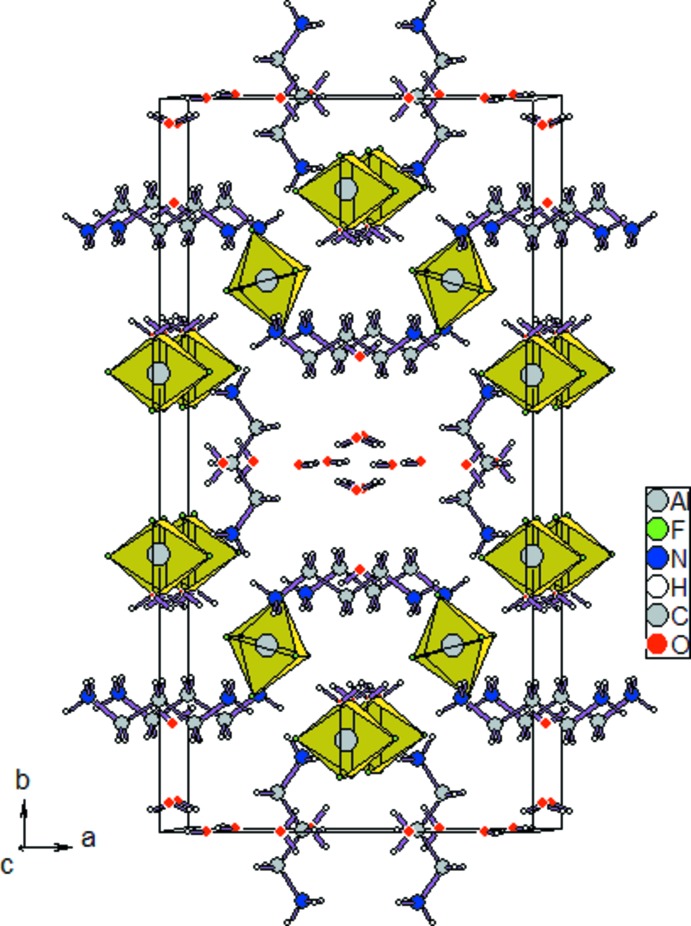
The crystal packing of the title compound, viewed approximately along [001].

Each [AlF_4_(H_2_O)_2_] octa­hedron is linked *via* N—H⋯F or O—H⋯F hydrogen bonds (Table 1 ▶) to one type of the organic cations (Fig. 2 ▶), with the formation of infinite chains parallel to the *a* axis. These chains are linked to each other by the AlF_6_^3−^ dications and form infinite (H_2_dap)[AlF_4_(H_2_O)_2_] layers parallel to the *ac* plane (Fig. 3 ▶) . These layers are connected by the second organic cations and form a three-dimensional framework showing cavities, which are filled with the lattice water mol­ecules.

## Database survey   

In the Cambridge Structural Database (Version 5.35; Groom & Allen, 2014 ▶) numerous *Class I* fluorido­aluminates with isolated (poly)anions or extended 1D inorganic chains, 2D inorganic layers or 3D networks are mentioned. Eight compounds with AlF_6_^3−^ anions exist (Grottel *et al.*, 1992 ▶; Rother *et al.*, 1996 ▶, 1998 ▶; Touret *et al.*, 2001 ▶; Adil *et al.*, 2009 ▶; Bentrup *et al.*, 1996 ▶) and seven compounds containing the AlF_5_(H_2_O)^2−^ anion (Cadiau *et al.*, 2008 ▶; Petrosyants *et al.*, 1997 ▶; Schröder *et al.*, 1993 ▶). However, to our knowledge, no fluorido­aluminate hybrid compounds containing both the AlF_6_^3−^ and AlF_5_(H_2_O)^2−^ anions have been reported.

## Synthesis and crystallization   

The title compound was prepared from a starting mixture of AlF_3_ (0.5 g) in 40% HF (1.5 ml) and ethanol (5 ml). 1,3-Di­amino­propane (0.54 ml) was added and mild hydro­thermal conditions (463 K) were applied in a Teflon-lined autoclave (25 ml). The resulting product was washed with ethanol and dried in air giving colourless single crystals.

## Refinement   

Crystal data, data collection and structure refinement details are summarized in Table 2 ▶. The H atoms of the NH_3_ and CH_2_ groups of the organic mol­ecule were fixed geometrically [N—H = 0.89 (1) and C—H = 0.97 (1) Å with *U*_iso_(H) = 1.2*U*_eq_(N,C)]. All H atoms of the water mol­ecules were located from a Fourier difference map. The O—H distances and H—O—H angles were fixed [O—H = 0.84 (1) and H⋯H = 1.34 (1) Å with *U*_iso_(H) =1.5*U*_eq_(O)]. The water mol­ecule O*W*5 is disordered over two positions with the occupanies fixed to 0.5.

## Supplementary Material

Crystal structure: contains datablock(s) I. DOI: 10.1107/S1600536814024155/cv5471sup1.cif

Structure factors: contains datablock(s) I. DOI: 10.1107/S1600536814024155/cv5471Isup2.hkl

CCDC reference: 1032262

Additional supporting information:  crystallographic information; 3D view; checkCIF report

## Figures and Tables

**Table 1:** Hydrogen-bond geometry (, )

*D*H*A*	*D*H	H*A*	*D* *A*	*D*H*A*
N1H1*A*F2	0.89	1.79	2.679(6)	177
N1H1*B*F5^i^	0.89	1.99	2.846(5)	161
N1H1*C*F5^ii^	0.89	1.99	2.846(5)	161
N2H2*A*F3	0.89	1.84	2.722(6)	173
N2H2*B*F1^iii^	0.89	2.00	2.841(5)	158
Ow1H1F5^iv^	0.83(4)	1.74(4)	2.569(5)	178
Ow2H2F3	0.84(4)	2.10(4)	2.880(5)	178
Ow4H4Ow5^v^	0.84(4)	2.13	2.910(5)	154

Symmetry codes: (i) 
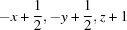
; (ii) 
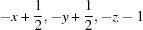
; (iii) 
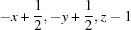
; (iv) 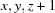; (v) 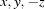.

**Table 2:** Experimental details

Crystal data	
Chemical formula	(C_3_H_12_N_2_)_2_[AlF_6_][AlF_4_(H_2_O)_2_]4H_2_O
*M*_r_	504.35
Crystal system, space group	Orthorhombic, *C* *m* *m* *m*
Temperature (K)	293
*a*, *b*, *c* ()	12.975(5), 25.115(9), 6.452(9)
*V* (^3^)	2103(3)
*Z*	4
Radiation type	Mo *K*
(mm^1^)	0.26
Crystal size (mm)	0.24 0.12 0.05

Data collection	
Diffractometer	Siemens AED2
No. of measured, independent and observed [*I* > 2(*I*)] reflections	1371, 1371, 939
(sin /)_max_ (^1^)	0.649

Refinement	
*R*[*F* ^2^ > 2(*F* ^2^)], *wR*(*F* ^2^), *S*	0.047, 0.148, 1.12
No. of reflections	1371
No. of parameters	105
No. of restraints	10
H-atom treatment	H atoms treated by a mixture of independent and constrained refinement
_max_, _min_ (e ^3^)	0.46, 0.48

Computer programs: *STADI4* and *X-RED* (Stoe Cie, 2002 ▶), *SHELXS97* (Sheldrick, 2008 ▶), *SHELXL97* (Sheldrick, 2008 ▶) within *WinGX* (Farrugia, 2012 ▶), *DIAMOND* (Brandenburg, 2006 ▶) and *publCIF* (Westrip, 2010 ▶).

## supplementary crystallographic information

### Crystal data

**Table:**

(C_3_H_12_N_2_)_2_[AlF_6_][AlF_4_(H_2_O)_2_]·4H_2_O	*F*(000) = 1056
*M**_r_* = 504.35	*D*_x_ = 1.593 Mg m^−^^3^
Orthorhombic, *C**m**m**m*	Mo *K*α radiation, λ = 0.71073 Å
Hall symbol: -C 2 2	Cell parameters from 32 reflections
*a* = 12.975 (5) Å	θ = 2–27.5°
*b* = 25.115 (9) Å	µ = 0.26 mm^−^^1^
*c* = 6.452 (9) Å	*T* = 293 K
*V* = 2103 (3) Å^3^	Platelets, colourless
*Z* = 4	0.24 × 0.12 × 0.05 mm

### Data collection

**Table:**

Siemens AED2 diffractometer	*R*_int_ = 0.000
Radiation source: fine-focus sealed tube	θ_max_ = 27.5°, θ_min_ = 1.6°
Graphite monochromator	*h* = 0→16
2θ/ω scan	*k* = 0→32
1371 measured reflections	*l* = 0→8
1371 independent reflections	3 standard reflections every 120 min
939 reflections with *I* > 2σ(*I*)	intensity decay: 4%

### Refinement

**Table:**

Refinement on *F*^2^	Secondary atom site location: difference Fourier map
Least-squares matrix: full	Hydrogen site location: inferred from neighbouring sites
*R*[*F*^2^ > 2σ(*F*^2^)] = 0.047	H atoms treated by a mixture of independent and constrained refinement
*wR*(*F*^2^) = 0.148	*w* = 1/[σ^2^(*F*_o_^2^) + (0.0452*P*)^2^ + 2.9098*P*] where *P* = (*F*_o_^2^ + 2*F*_c_^2^)/3
*S* = 1.12	(Δ/σ)_max_ < 0.001
1371 reflections	Δρ_max_ = 0.46 e Å^−^^3^
105 parameters	Δρ_min_ = −0.48 e Å^−^^3^
10 restraints	Extinction correction: *WinGX* (Farrugia, 2012), Fc^*^=kFc[1+0.001xFc^2^λ^3^/sin(2θ)]^-1/4^
Primary atom site location: structure-invariant direct methods	Extinction coefficient: 0.0022 (7)

### Special details

**Table:**

Geometry. All e.s.d.'s (except the e.s.d. in the dihedral angle between two l.s. planes) are estimated using the full covariance matrix. The cell e.s.d.'s are taken into account individually in the estimation of e.s.d.'s in distances, angles and torsion angles; correlations between e.s.d.'s in cell parameters are only used when they are defined by crystal symmetry. An approximate (isotropic) treatment of cell e.s.d.'s is used for estimating e.s.d.'s involving l.s. planes.
Refinement. Refinement of *F*^2^ against ALL reflections. The weighted *R*-factor *wR* and goodness of fit *S* are based on *F*^2^, conventional *R*-factors *R* are based on *F*, with *F* set to zero for negative *F*^2^. The threshold expression of *F*^2^ > σ(*F*^2^) is used only for calculating *R*-factors(gt) *etc*. and is not relevant to the choice of reflections for refinement. *R*-factors based on *F*^2^ are statistically about twice as large as those based on *F*, and *R*- factors based on ALL data will be even larger.

### Fractional atomic coordinates and isotropic or equivalent isotropic displacement parameters (Å^2^)

**Table:**

	*x*	*y*	*z*	*U*_iso_*/*U*_eq_	Occ. (<1)
Al1	0.0000	0.37606 (7)	0.0000	0.0234 (4)	
Al2	0.2500	0.2500	−0.5000	0.0245 (4)	
F1	0.0000	0.42435 (10)	0.2015 (4)	0.0382 (6)	
F2	0.13615 (17)	0.37342 (11)	0.0000	0.0374 (6)	
F3	0.2107 (2)	0.18091 (10)	−0.5000	0.0493 (8)	
F5	0.15743 (14)	0.26362 (8)	−0.7007 (3)	0.0447 (5)	
N1	0.3125 (2)	0.31814 (15)	0.0000	0.0325 (8)	
H1A	0.2551	0.3375	0.0000	0.049*	
H1B	0.3141	0.2977	0.1126	0.049*	0.50
H1C	0.3141	0.2977	−0.1126	0.049*	0.50
N2	0.3447 (3)	0.09755 (14)	−0.5000	0.0331 (8)	
H2A	0.3055	0.1266	−0.5000	0.050*	
H2B	0.3843	0.0974	−0.6126	0.050*	0.50
H2C	0.3843	0.0974	−0.3874	0.050*	0.50
C1	0.4029 (3)	0.35390 (18)	0.0000	0.0375 (11)	
H1D	0.4014	0.3765	0.1219	0.045*	0.50
H1E	0.4014	0.3765	−0.1219	0.045*	0.50
C2	0.5000	0.3205 (2)	0.0000	0.0336 (14)	
H2D	0.5000	0.2977	0.1215	0.040*	0.50
H2E	0.5000	0.2977	−0.1215	0.040*	0.50
C3	0.2784 (3)	0.04975 (18)	−0.5000	0.0415 (11)	
H3A	0.2346	0.0501	−0.6217	0.050*	0.50
H3B	0.2346	0.0501	−0.3783	0.050*	0.50
C4	0.3432 (5)	0.0000	−0.5000	0.0427 (16)	
H4A	0.3872	0.0000	−0.6215	0.051*	0.50
H4B	0.3872	0.0000	−0.3785	0.051*	0.50
OW1	0.0000	0.32173 (12)	0.2146 (6)	0.0400 (8)	
OW2	0.0000	0.1449 (3)	−0.5000	0.080 (2)	
OW3	0.3272 (7)	0.0000	0.0000	0.104 (3)	
OW4	0.3732 (11)	0.5000	0.0000	0.200 (6)	
OW5	0.5000	0.5374 (6)	0.342 (2)	0.110 (4)	0.50
H1	0.0517 (4)	0.3034 (11)	0.241 (9)	0.165*	
H2	0.0517 (4)	0.1650 (8)	−0.5000	0.165*	
H3	0.3669 (15)	0.0267 (2)	0.0000	0.165*	
H4	0.4125 (17)	0.5000	−0.1039 (8)	0.165*	
H5	0.5516 (4)	0.526 (5)	0.408 (12)	0.165*	0.50

### Atomic displacement parameters (Å^2^)

**Table:**

	*U*^11^	*U*^22^	*U*^33^	*U*^12^	*U*^13^	*U*^23^
Al1	0.0187 (8)	0.0233 (8)	0.0282 (9)	0.000	0.000	0.000
Al2	0.0230 (8)	0.0257 (8)	0.0248 (8)	0.0074 (7)	0.000	0.000
F1	0.0469 (14)	0.0332 (13)	0.0346 (13)	0.000	0.000	−0.0076 (11)
F2	0.0190 (11)	0.0373 (14)	0.0560 (16)	−0.0036 (10)	0.000	0.000
F3	0.0445 (16)	0.0282 (14)	0.075 (2)	0.0027 (12)	0.000	0.000
F5	0.0370 (9)	0.0582 (13)	0.0388 (10)	0.0178 (8)	−0.0120 (8)	−0.0007 (9)
N1	0.0211 (16)	0.043 (2)	0.0338 (18)	0.0014 (15)	0.000	0.000
N2	0.0342 (19)	0.0262 (17)	0.039 (2)	0.0023 (15)	0.000	0.000
C1	0.027 (2)	0.030 (2)	0.056 (3)	0.0028 (17)	0.000	0.000
C2	0.020 (3)	0.029 (3)	0.053 (4)	0.000	0.000	0.000
C3	0.027 (2)	0.033 (2)	0.064 (3)	0.0040 (18)	0.000	0.000
C4	0.028 (3)	0.029 (3)	0.071 (5)	0.000	0.000	0.000
OW1	0.0266 (14)	0.0385 (16)	0.055 (2)	0.000	0.000	0.0203 (16)
OW2	0.065 (4)	0.104 (6)	0.070 (4)	0.000	0.000	0.000
OW3	0.121 (7)	0.090 (6)	0.102 (6)	0.000	0.000	0.000
OW4	0.209 (14)	0.204 (17)	0.188 (13)	0.000	0.000	0.000
OW5	0.101 (8)	0.110 (9)	0.119 (10)	0.000	0.000	0.032 (8)

### Geometric parameters (Å, º)

**Table:**

Al1—F2^i^	1.768 (2)	N2—H2C	0.8900
Al1—F2	1.768 (2)	C1—C2	1.514 (5)
Al1—F1	1.778 (3)	C1—H1D	0.9700
Al1—F1^ii^	1.778 (3)	C1—H1E	0.9700
Al1—OW1	1.944 (4)	C2—C1^vi^	1.514 (5)
Al1—OW1^ii^	1.944 (4)	C2—H2D	0.9700
Al2—F5^iii^	1.799 (2)	C2—H2E	0.9700
Al2—F5	1.799 (2)	C3—C4	1.505 (5)
Al2—F5^iv^	1.799 (2)	C3—H3A	0.9700
Al2—F5^v^	1.799 (2)	C3—H3B	0.9700
Al2—F3	1.809 (3)	C4—C3^vii^	1.505 (5)
Al2—F3^iii^	1.809 (3)	C4—H4A	0.9700
N1—C1	1.476 (6)	C4—H4B	0.9700
N1—H1A	0.8900	OW1—H1	0.831 (10)
N1—H1B	0.8900	OW2—H2	0.840 (10)
N1—H1C	0.8900	OW3—H3	0.846 (10)
N2—C3	1.477 (6)	OW4—H4	0.842 (10)
N2—H2A	0.8900	OW5—H5	0.840 (10)
N2—H2B	0.8900		
			
F2^i^—Al1—F2	175.7 (2)	H1A—N1—H1C	109.5
F2^i^—Al1—F1	91.47 (7)	H1B—N1—H1C	109.5
F2—Al1—F1	91.47 (7)	C3—N2—H2A	109.5
F2^i^—Al1—F1^ii^	91.47 (7)	C3—N2—H2B	109.5
F2—Al1—F1^ii^	91.47 (7)	H2A—N2—H2B	109.5
F1—Al1—F1^ii^	94.0 (2)	C3—N2—H2C	109.5
F2^i^—Al1—OW1	88.49 (7)	H2A—N2—H2C	109.5
F2—Al1—OW1	88.49 (7)	H2B—N2—H2C	109.5
F1—Al1—OW1	87.59 (16)	N1—C1—C2	108.9 (4)
F1^ii^—Al1—OW1	178.44 (16)	N1—C1—H1D	109.9
F2^i^—Al1—OW1^ii^	88.49 (7)	C2—C1—H1D	109.9
F2—Al1—OW1^ii^	88.49 (7)	N1—C1—H1E	109.9
F1—Al1—OW1^ii^	178.44 (16)	C2—C1—H1E	109.9
F1^ii^—Al1—OW1^ii^	87.60 (16)	H1D—C1—H1E	108.3
OW1—Al1—OW1^ii^	90.8 (2)	C1^vi^—C2—C1	112.7 (5)
F5^iii^—Al2—F5	180.00 (14)	C1^vi^—C2—H2D	109.1
F5^iii^—Al2—F5^iv^	87.92 (14)	C1—C2—H2D	109.1
F5—Al2—F5^iv^	92.08 (14)	C1^vi^—C2—H2E	109.1
F5^iii^—Al2—F5^v^	92.08 (14)	C1—C2—H2E	109.1
F5—Al2—F5^v^	87.92 (14)	H2D—C2—H2E	107.8
F5^iv^—Al2—F5^v^	180.00 (13)	N2—C3—C4	110.5 (4)
F5^iii^—Al2—F3	90.34 (9)	N2—C3—H3A	109.6
F5—Al2—F3	89.66 (9)	C4—C3—H3A	109.6
F5^iv^—Al2—F3	89.66 (9)	N2—C3—H3B	109.6
F5^v^—Al2—F3	90.34 (9)	C4—C3—H3B	109.6
F5^iii^—Al2—F3^iii^	89.66 (9)	H3A—C3—H3B	108.1
F5—Al2—F3^iii^	90.34 (9)	C3^vii^—C4—C3	112.2 (5)
F5^iv^—Al2—F3^iii^	90.34 (9)	C3^vii^—C4—H4A	109.2
F5^v^—Al2—F3^iii^	89.66 (9)	C3—C4—H4A	109.2
F3—Al2—F3^iii^	180.0	C3^vii^—C4—H4B	109.2
C1—N1—H1A	109.5	C3—C4—H4B	109.2
C1—N1—H1B	109.5	H4A—C4—H4B	107.9
H1A—N1—H1B	109.5	Al1—OW1—H1	122 (2)
C1—N1—H1C	109.5		

Symmetry codes: (i) −*x*, *y*, *z*; (ii) *x*, *y*, −*z*; (iii) −*x*+1/2, −*y*+1/2, −*z*−1; (iv) *x*, *y*, −*z*−1; (v) −*x*+1/2, −*y*+1/2, *z*; (vi) −*x*+1, *y*, *z*; (vii) *x*, −*y*, −*z*−1.

### Hydrogen-bond geometry (Å, º)

**Table:**

*D*—H···*A*	*D*—H	H···*A*	*D*···*A*	*D*—H···*A*
N1—H1*A*···F2	0.89	1.79	2.679 (6)	177
N1—H1*B*···F5^viii^	0.89	1.99	2.846 (5)	161
N1—H1*C*···F5^iii^	0.89	1.99	2.846 (5)	161
N2—H2*A*···F3	0.89	1.84	2.722 (6)	173
N2—H2*B*···F1^ix^	0.89	2.00	2.841 (5)	158
Ow1—H1···F5^x^	0.83 (4)	1.74 (4)	2.569 (5)	178
Ow2—H2···F3	0.84 (4)	2.10 (4)	2.880 (5)	178
Ow4—H4···Ow5^ii^	0.84 (4)	2.13	2.910 (5)	154

Symmetry codes: (ii) *x*, *y*, −*z*; (iii) −*x*+1/2, −*y*+1/2, −*z*−1; (viii) −*x*+1/2, −*y*+1/2, *z*+1; (ix) −*x*+1/2, −*y*+1/2, *z*−1; (x) *x*, *y*, *z*+1.
